# Association of Nutritional Risk Index With Postoperative Pain Outcomes in Elderly Patients Undergoing Gastrointestinal Surgeries: A Retrospective Cohort Study

**DOI:** 10.3389/fmed.2021.535627

**Published:** 2021-09-09

**Authors:** Hua Zheng, Guangyou Duan, Shiqian Shen, Xianwei Zhang

**Affiliations:** ^1^Department of Anesthesiology, Tongji Hospital, Tongji Medical College, Huazhong University of Science and Technology, Wuhan, China; ^2^Department of Anesthesiology, Second Affiliated Hospital, Chongqing Medical University, Chongqing, China; ^3^MGH Center for Translational Pain Research, Department of Anesthesia, Critical Care and Pain Medicine, Massachusetts General Hospital and Harvard Medical School, Boston, MA, United States

**Keywords:** geriatric nutritional risk index, postoperative pain, postoperative inadequate analgesia, gastrointestinal surgeries, elderly patients

## Abstract

**Background:** Malnutrition is a major health problem, which is common in hospitalized elderly patients and is associated with an increased risk of morbidity and mortality. However, studies on malnutrition and its effect on postoperative pain outcomes in elderly patients have been largely neglected. Here we investigated the relationship between nutritional risk and postoperative pain outcomes in elderly patients.

**Methods:** Between April 1, 2012, and August 31, 2015, 734 elderly patients (≥65 years) who underwent gastrointestinal surgeries were recruited and assigned into two groups according to geriatric nutritional risk index (GNRI). All patients received standard anesthesia procedures and postoperative patient-controlled analgesia for 48 h. The preoperative epidemiology data and postoperative outcome data including pain intensities at rest and movement, the cumulative consumption of analgesics and its common side effects were recorded.

**Results:** The total number of patients with high nutritional risk (GNRI < 92) was 533 out of 734 (72.62%). When compared with low nutritional risk individuals (GNRI ≥ 92), the incidence of inadequate analgesia was significantly higher in elderly patients with GNRI < 92 at different time points. In addition, the cumulative consumption of analgesics was also significantly higher in elderly patients with GNRI < 92 at 0–6 h postoperatively. Through logistic regression analysis, high nutritional risk (OR = 3.113, 95% CI: 1.661–5.834, *P* < 0.001) and female gender (OR = 0.606, 95% CI: 0.394–0.932, *P* = 0.023) were identified as significant predictors for postoperative inadequate analgesia. Further sensitivity analyses showed high nutritional risk as a predictor for postoperative inadequate analgesia was more prominent in female patients and early elderly patients. Moreover, 88 was determined as an optimal cut-off value of GNRI for postoperative inadequate analgesia using receiver operating characteristic curve analysis.

**Conclusion:** High nutritional risk is associated with poor postoperative pain outcomes in gastrointestinal elderly patients. Preoperative nutritional evaluation using simple nutritional screening instruments (e.g., GNRI) with the new suggested cut-off value (GNRI = 88) might be included as a standard procedure in routine clinical practice among these patients for postoperative analgesia.

## Introduction

Population aging is a worldwide phenomenon. It is predicted that persons over 65 years will compose 30% of the total population by the year 2050 (1). Although these aged individuals are expected to increase demand for surgical treatments (2), management of postoperative pain in elderly patients continues to be a major challenge. It is reported that ~50–75% of elderly patients experience inadequate postoperative pain relief (3). The under treatment of postoperative pain is associated with serious negative consequences, including increased risk of myocardial, thromboembolic or pulmonary complications, impaired rehabilitation, increased length of hospital stay, increased risk of persistent postoperative pain and elevated mortality rate (4).

The failure to provide appropriate postoperative analgesia in elderly patients is multifactorial. One of the common reasons is inadequate knowledge about physiological or pathophysiological changes and their effects on postoperative pain management in elderly patients (5). As a result of aging processes, elderly patients are at higher risk of malnutrition due to decreased gastric secretions and intestinal motility (6), which has been proven to predict morbidity and mortality among older hospitalized patients (7, 8). However, thus far, there have been no studies examining the relationship between nutritional risk and postoperative pain outcomes in elderly patients.

Geriatric nutritional risk index (GNRI) is a nutritional screening and assessment tool created to predict nutrition-related complications in hospitalized elderly patients (9). A lower value of GNRI indicates a higher nutrition-related risk. In a previous study in elderly patients with acute heart failure, it was found that GNRI < 92 is associated with poor clinical outcomes (10). In another 3-year follow-up study, a GNRI <92 was reported to associated with higher mortality and suggested as a profitable clinical trigger for routine nutritional treatment (11). However, GNRI has not been used to predict postoperative pain outcomes and its optimal cut-off value is unclear.

Accordingly, the main aim of this study was determining whether nutritional risk was associated with postoperative pain outcomes using GNRI and the optimal cut-off value of the GNRI for postoperative inadequate analgesia. Furthermore, we evaluate the influence of nutritional status on postoperative pain outcomes in gastrointestinal elderly patients receiving patient-controlled intravenous analgesia.

## Materials and Methods

### Study Design and Data Sources

Institutional Review Board (IRB) approval for this retrospective cohort study was granted through the Ethic Committee of Tongji Hospital, Tongji Medical College, Huazhong University of Science and Technology (TJ-IRB20190403). The requirement for informed consent from participants was waived under the regulations of IRB. Demographic data (gender, age, weight, height, etc.), preoperative data (comorbidity, serum albumin, etc.) and process data (surgical types, surgical methods, anesthesia techniques, intraoperative medication, analgesia technique, etc.) presented in the current study were extracted from the patients' electronic medical records. Outcome data (pain intensities at rest and movement, cumulative analgesics consumption, side effects of analgesics, etc.) were collected by an acute pain service group at different time points postoperatively (12–14). Approximately 82% of the patients undergoing gastrointestinal surgeries were included. All data were assessed and edited by two authors (HZ, GD). If a missing data or an extreme value occurred, the relevant raw data were double checked. Participant's name or other form of identification was deleted before analysis. The reporting of this study followed the STROBE (strengthening the reporting of observational studies in epidemiology) (15) and RECORD (reporting of studies conducted using observational routinely collected health data) (16) guidelines.

### Participants

Between April 1, 2012, and August 31, 2015, elderly patients (≥65 years) who underwent gastrointestinal surgeries were screened. Inclusion criteria were receiving general anesthesia and postoperative patient-controlled analgesia for 48 h. Exclusion criteria were receiving regional anesthesia, undergone repeat surgery during hospitalization and missing data for any variable.

### Exposure of Interest

The exposure of interest in this study was high nutritional risk. The risk of nutritional status was assessed by the geriatric nutritional risk index (GNRI), which was designed specifically for the hospitalized elderly patients (9). The GNRI was calculated based on the patient's weight, height and serum albumin as follows: GNRI = [1.489 × albumin (g/L)] + [41.7 × (weight/WLo)]. The WLo is the ideal weight and was calculated using the Lorentz formula as WLo = 0.75 × height (cm) − 62.5 for men and WLo = 0.60 × height (cm) − 40 for women (17). When weight exceeded ideal weight, the ration of weight/WLo was set to 1. Similar to previous studies, the GNRI of 92 was taken as an original cut-off value (10, 11, 18).

### Perioperative Pain Management and Outcome Measures

All patients were treated according to the standard procedures at Tongji Hospital. In general, anesthesia induction was performed using 0.3–0.6 μg/kg sufentanil, 0.1–0.2 mg/kg cisatracurium and 1.5–2.5 mg/kg propofol. Anesthesia was maintained with a combination of sevoflurane (1.0–2.0%), remifentanil (0.2–0.4 μg·kg^−1^·min^−1^) and propofol (6–10 mg·kg^−1^·h^−1^). At 15 min prior to the surgery, patients without contraindication were given nonsteroidal anti-inflammatory drugs (NSAIDs, 40 mg parecoxib sodium) and prophylactic antiemetics (dexamethasone 4 mg and/or tropisetron hydrochloride 2 mg). Immediately after surgery, patient-controlled intravenous analgesia (PCIA) was started with 0.7 μg/mL sufentanil and 4 mg/mL tramadol using a PCIA pump (BCM, BCDB-150, Shanghai, China). The pump was programmed to use a background infusion at 1–2.5 mL/h, a bolus dose of 1 mL, a lockout interval of 10 min, and a dose limit of 12 mL/h.

Patient outcome data were collected by the acute pain service group at 0–6, 18–24, and 42–48 h postoperatively. Pain intensities at rest and movement were assessed using a 100-mm visual analog scale (VAS, 100 being worst pain imaginable). In addition, cumulative PCIA consumption and the common side effects including postoperative nausea and vomiting, respiratory depression, abdominal distention, pruritus, urinary retention, and dizziness were also recorded. Pain trigger for rescue analgesia was VAS ≥ 40, which was defined as postoperative inadequate analgesia (13, 19). Under these circumstances, Patients without contraindication were given parecoxib sodium and PCIA parameters were upregulated. The primary outcome we used here was postoperative inadequate analgesia. As secondary outcome we investigated cumulative PCIA consumption and the side effects of PCIA.

### Statistical Analysis

In the database, about 8.9% of data were missed. The missing data arose mainly in variable “postoperative cumulative PCIA consumption” but not in variables included in the regression analysis. Thus, traditional statistical analyses were performed and imputation analyses were not considered. Participants with any missing data were excluded from analysis. Demographic data (e.g., age, weight and height) and postoperative cumulative PCIA consumption were presented as median (interquartile range). These data did not pass the Shapiro-Wilk test for normal distribution and were analyzed by the Mann–Whitney *U* test. Body mass index (BMI) were presented as mean (± standard deviation) and compared using the *t* test. Dichotomous data (e.g., gender and postoperative inadequate analgesia) were expressed as absolute number (and %) and significance was calculated with the chi-square test. To evaluate the role of the preoperative factors in the prediction of postoperative inadequate analgesia during the entire 0–48 h period, a forward stepwise logistic regression model was applied. Gender, age, BMI, Charlson Comorbidity Index (CCI) score, American society of anesthesiologists (ASA) score, surgical types (gastric or intestinal), surgical methods (endoscopic or non-endoscopic), intraoperative medication and the GNRI were included in the model. Given gender and age were reported to be risk factors for postoperative inadequate analgesia in our previous study (20), sensitivity analyses were further performed in female and male subgroups, as well as in early elderly (age <75 years) and late elderly (age ≥75 years) subgroups. A receiver operating characteristic (ROC) curve analysis was used to determine an optimal cut-off value of GNRI for postoperative inadequate analgesia. A *P* < 0.05 was considered statistically significant. All of the statistical analyses were performed with SPSS software (version 17.0, SPSS Inc., Chicago, IL, USA).

## Results

### Characteristics of the Patients

**Figure 1:**
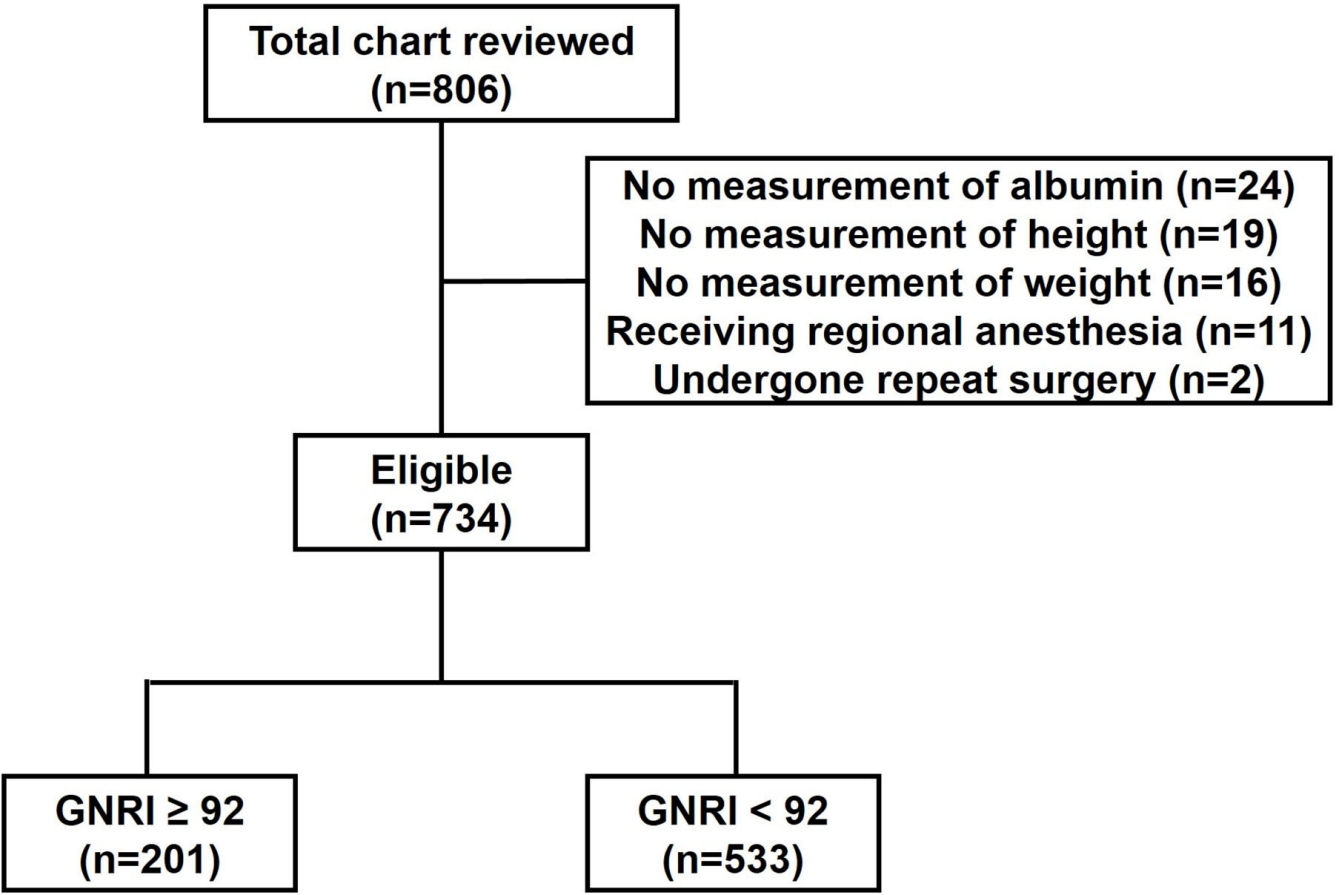
Study flow. GNRI, geriatric nutritional risk index.

During the study period, a total of 806 patients were reviewed. Of them, 59 patients with missing data for any variable, 11 patients receiving regional anesthesia and two patients undergone repeat surgery were excluded. Finally, 734 patients were included in the analysis (Figure 1).

Taking GNRI of 92 as a cut-off point, the total number of patients with high nutritional risk (GNRI < 92) was 533 out of 734 (72.62%). Demographic characteristics of patients were summarized in Table 1. The median age of the whole population was 69 (67–74), whereas patients with GNRI < 92 were slightly older than patients with GNRI ≥ 92 (70 (67–74) vs. 68 (66–73); *p* = 0.002). Weight (57 (50–65) vs. 63 (58–71); *p* < 0.001), height (1.64 (1.58–1.70) vs. 1.65 (1.60–1.70); *p* = 0.032) and BMI (21.64 ± 3.60 vs. 23.70 ± 2.73; *p* < 0.001) were lower in patients with GNRI < 92 compared to patients with GNRI ≥ 92. In addition, less endoscopic surgeries were performed in patients with GNRI < 92 than patients with GNRI ≥ 92 (74.48 vs. 82.09%; *p* < 0.030). Gender, CCI scores, ASA scores, surgical types and intraoperative medication including dexamethasone, parecoxib and sufentanil were not different among groups.

**Table 1:** Comparison of patient characteristics in different nutritional risk groups as classified by the GNRI.

**Characteristic**	**Total (*n* = 734)**	**GNRI ≥ 92 (*n* = 201)**	**GNRI < 92 (*n* = 533)**	***P* value**
Gender (male/female)	472 (64.31%)/262 (35.69%)	132 (65.67%)/69 (34.33%)	340 (63.79%)/193 (36.21%)	0.635
Age (years)	69 (67–74)	68 (66–73)	70 (67–74)	0.002
Weight (kg)	60 (51–67)	63 (58–71)	57 (50–65)	<0.001
Height (m)	1.65 (1.59–1.70)	1.65 (1.60–1.70)	1.64 (1.58–1.70)	0.032
BMI (kg/m^2^)	22.20 ± 3.51	23.70 ± 2.73	21.64 ± 3.60	<0.001
CCI score (0/1/2/ ≥ 3)	276 (37.60%)/263 (35.83%)/100 (13.62%)/ 95 (12.94%)	87 (43.28%)/67 (33.33%)/28 (13.93%)/ 19 (9.45%)	189 (35.46%)/196 (36.77%)/72 (13.51%)/ 76 (14.26%)	0.140
ASA score (I/II/III/IV)	262 (35.70%)/287 (39.10%)/114 (15.53%)/ 71 (9.67%)	82 (40.80%)/74 (36.82%)/26 (12.94%)/ 19 (9.45%)	180 (33.77%)/213 (39.96%)/88 (16.51%)/ 52 (9.76%)	0.308
Surgical types (gastric/intestinal)	415 (56.54%)/319 (43.46%)	118 (58.71%)/83 (41.29%)	297 (55.72%)/236 (44.28%)	0.467
Surgical methods (E/non-E)	562 (76.57%)/172 (23.43%)	165 (82.09%)/36 (17.91%)	397 (74.48%)/136 (25.52%)	0.030
**Intraoperative medication**				
Dexamethasone (yes/no)	415 (56.54%)/319 (43.46%)	106 (52.74%)/95 (47.26%)	309 (57.97%)/224 (42.03%)	0.202
Parecoxib (yes/no)	421 (57.36%)/313 (42.64%)	125 (62.19%)/76 (37.81%)	296 (55.53%)/237 (44.47%)	0.104
Sufentanil (μg)	25 (20–30)	25 (20–30)	25 (20–30)	0.090

*Results are presented as median (interquartile range), mean (± standard deviation) or relative numbers [n (%)]. Groups were compared by using Mann-Whitney U test, t test or chi-square test. GNRI, geriatric nutritional risk index; BMI, body mass index; CCI, Charlson Comorbidity Index; ASA, American Society of Anesthesiologists; E, endoscopic*.

### Postoperative Pain Outcomes in Patients With Different Nutritional Risk

Firstly, we compared postoperative pain outcomes in different nutritional risk groups as classified by the GNRI, which are shown in Table 2. At rest, the incidence of inadequate analgesia of the whole population was 13.62% at 0–6 h, 9.54% at 18–24 h and 3.95% at 42–48 h postoperatively; the patients with GNRI < 92 had higher levels of inadequate analgesia at 0–6 h (16.51 vs. 5.97%; *p* < 0.001), 18–24 h (11.82 vs. 3.48%; *p* = 0.001), but not 42–48 h postoperatively, in comparison with the patients with GNRI ≥ 92. On movement, 20.44% patients at 0–6 h, 14.17% patients at 18–24 h and 8.99% patients at 42–48 h postoperatively presented inadequate analgesia; the incidence of inadequate analgesia in patients with GNRI < 92 was higher than the patients with GNRI ≥ 92 at 0–6 h (24.02 vs. 10.95%; *p* < 0.001), 18–24 h (16.51 vs. 7.96%; *p* = 0.003) and 42–48 h (10.51 vs. 4.98%; *p* = 0.019) postoperatively.

**Table 2:** Comparison of postoperative pain outcomes in different nutritional risk groups as classified by the GNRI.

**Outcome**	**Total (*n* = 734)**	**GNRI ≥ 92 (*n* = 201)**	**GNRI < 92 (*n* = 533)**	***P* value**
Postoperative inadequate analgesia at rest				
0–6 h	100 (13.62%)	12 (5.97%)	88 (16.51%)	<0.001
18–24 h	70 (9.54%)	7 (3.48%)	63 (11.82%)	0.001
42–48 h	29 (3.95%)	4 (1.99%)	25 (4.69%)	0.094
Postoperative inadequate analgesia on movement				
0–6 h	150 (20.44%)	22 (10.95%)	128 (24.02%)	<0.001
18–24 h	104 (14.17%)	16 (7.96%)	88 (16.51%)	0.003
42–48 h	66 (8.99%)	10 (4.98%)	56 (10.51%)	0.019
Postoperative cumulative PCIA consumption (ml/kg)				
0–6 h	0.15 (0.08–0.26)	0.15 (0.07–0.22)	0.16 (0.08–0.27)	0.036
18–24 h	0.44 (0.27–0.67)	0.41 (0.28–0.61)	0.46 (0.27–0.70)	0.103
42–48 h	0.64 (0.41–0.96)	0.60 (0.43–0.92)	0.64 (0.40–1.00)	0.246
Side effects of PCIA				
Nausea/vomiting	101 (13.76%)	21 (10.45%)	80 (15.01%)	0.110
Dizziness	36 (4.90%)	12 (5.97%)	24 (4.50%)	0.412
Abdominal distension	29 (3.95%)	6 (2.99%)	23 (4.32%)	0.409
Urinary retention	6 (0.82%)	2 (1.00%)	4 (0.75%)	0.743
Pruritus	4 (0.54%)	1 (0.50%)	3 (0.56%)	0.915
Respiratory depression	2 (0.27%)	0 (0.00%)	2 (0.38%)	0.384

*Results are presented as median (interquartile range) or relative numbers [n (%)]. Groups were compared by using Mann-Whitney U test or chi-square test. GNRI, geriatric nutritional risk index; PCA, Patient-controlled analgesia*.

In all patients the postoperative cumulative PCIA consumption was 0.15 (0.08–0.26) mL/kg at 0–6 h, 0.44 (0.27–0.67) mL/kg at 18–24 k and 0.64 (0.41–0.96) mL/kg at 42–48 h postoperatively. Patients with GNRI < 92 received significantly more cumulative PCIA consumption compared to patients with GNRI ≥ 92 at 0–6 h (0.16 (0.08–0.27) vs. 0.15 (0.07–0.22) mL/kg; *p* = 0.036), but not at 18–24 h and 42–48 h postoperatively.

During 48 h follow-up, postoperative nausea and vomiting was the most common side effect of PCIA with the incidence of 13.76%, followed by dizziness (4.90%), abdominal distention (3.95%), urinary retention (0.82%), pruritus (0.54%) and respiratory depression (0.27%). No significant differences were noted regarding the side effects of PCIA among the two groups.

### Association of Nutritional Risk With Postoperative Inadequate Analgesia

Secondly, we performed a logistic regression analyses in order to identify possible predictors for postoperative inadequate analgesia at rest during 48 h follow-up. The results showed this overall model was significant (P <0.001). As presented in Table 3, high nutritional risk (OR = 3.113, 95% CI: 1.661–5.834, *P* < 0.001) but not age (*P* = 0.172), BMI (*P* = 0.888), CCI score (*P* = 0.539), ASA score (*P* = 0.701), surgical types (*P* = 0.814), surgical methods (*P* = 0.859) and intraoperative use of dexamethasone (*P* = 0.698), parecoxib (*P* = 0.282) and sufentanil (*P* = 0.366) was identified as a significant predictor for postoperative inadequate analgesia, indicating that the probability of occurrence of postoperative inadequate analgesia in patients with GNRI < 92 was higher than patients with GNRI ≥ 92. In addition, The OR for female gender was 0.606 (95% CI: 0.394–0.932, *P* = 0.023), indicating that, compared with male patients, female patients would have a higher risk to report postoperative inadequate analgesia. Furthermore, sensitivity analyses showed high nutritional risk as a predictor for postoperative inadequate analgesia was more prominent in female patients (OR = 6.349, 95% CI: 1.901–21.201, *P* = 0.003) and early elderly patients (OR = 4.302, 95% CI: 1.932–9.579, *P* < 0.001). Collectively, high nutritional risk was associated with postoperative inadequate analgesia in elderly patients after gastrointestinal surgeries, especially in female individuals and early elderly patients.

**Table 3:** Logistic regression analysis investigating possible predictors for postoperative inadequate analgesia.

**Population**	**Predictors**	***P* value**	**OR**	**Lower 95%CI**	**Upper 95%CI**
Overall (*n* = 734)	Gender	0.023	0.606	0.394	0.932
Overall (*n* = 734)	GNRI	<0.001	3.113	1.661	5.834
Female (*n* = 262)	GNRI	0.003	6.349	1.901	21.201
Early elderly (Age <75 years, *n* = 587)	GNRI	<0.001	4.302	1.932	9.579

*GNRI, geriatric nutritional risk index; OR, odds ratio; CI, confidence interval*.

Given that GNRI was a predictor for postoperative inadequate analgesia, we used the ROC curve analysis to obtain the area under the curve and an optimal cut-off value of the GNRI. Area under ROC curve was 0.584 (95% CI: 0.531–0.637, *p* = 0.007). GNRI = 88 was determined as an optimal cut-off value with maximum discriminative power. Compared with the original cut-off value of 92, the new cut-off value of 88 had higher specificity (0.465 vs. 0.298) but lower sensitivity (0.730 vs. 0.880).

## Discussion

In this large sample of elderly patients after gastrointestinal surgery we showed that GNRI is a significant predictor for postoperative inadequate analgesia at rest during the first 48 h postoperatively. Elderly patients with lower GNRI values were at higher risk to experience inadequate postoperative pain relief than patients with higher GNRI values. Furthermore, we determined that 88 was an optimal cut-off value of GNRI for postoperative inadequate analgesia.

In the first part of the study, we determined the prevalence of nutrition-related risk according to an original GNRI cut-off value of 92. The results showed that majority (almost three quarters) of elderly patients in the current study were at high nutritional risk. A previous prospective cohort study reports the prevalence of severe and moderate risk of nutritional-related complication in hospitalized elderly patients is 41.2% (21). A recent retrospective study shows that 61.6% critical limb ischemia patients are at high nutritional risk (22). Another population-based survey in community-dwelling older persons finds that 69% of participants are at moderate to high nutritional risk (23). This variability is probably due to the differences of population, measurement instruments and cut-off values. Besides, our results showed that older gastrointestinal patients with high nutritional risk are not uncommon and the prevalence of high nutritional risk has always been underestimated. Thus, using of simple nutritional screening instruments (e.g., GNRI) should be included as a standard procedure in routine clinical practice (17).

Next, we compared patient characteristics and postoperative pain outcomes in different nutritional groups. As expected, we observed that patients with lower GNRI showed significantly higher values of age and lower values of weight, height and BMI. Interestingly, less patients with higher nutritional risk received endoscopic surgeries. The probable reason of this phenomenon might be the higher incidence of cardiopulmonary diseases in patients with higher nutritional risk, which was considered to be a relative contraindication to carbon dioxide pneumoperitoneum during endoscopic surgeries (24). Then, our results showed that the prevalence of postoperative inadequate analgesia at rest and movement was higher in patients with GNRI < 92 than patients with GNRI ≥ 92 at different time points. This is consist with a previous study, which shows that pain intensities was higher among patients in low nutritional status than normal patients (25). Another cross-sectional study also shows that the mean nutritional risk score is higher in patients with chronic musculoskeletal pain than patients without chronic musculoskeletal pain (23). Moreover, postoperative cumulative PCIA consumption was higher in patients with GNRI < 92 than patients with GNRI ≥ 92 in the current study. In another word, even the patients with GNRI < 92 received more analgesics, they still experienced severer postoperative pain. Collectively, high nutritional risk may lead to poor pain management in elderly patients after gastrointestinal surgeries and should not be ignored.

In the second part of the study, we explored whether high nutritional risk is a preoperative factor in the prediction of the primary outcome, postoperative inadequate analgesia. Through regression analysis, high nutritional risk was identified as having a negative effect on postoperative pain. This observation consists with the results of Takahashi et al. (25), who found a correlation between the Nutrition Risk Screening 2002 (NRS 2002) scores and the pain intensities. In community-dwelling older persons, nutritional risk was also reported as being independently associated with chronic musculoskeletal pain (23). The association between high nutritional risk and postoperative inadequate analgesia possibly refers to a systemic inflammatory response, which is triggered by undernutrition and might lead to CNS sensitization and amplification of pain through three pathways (26). Firstly, poor nutrition causes peripheral inflammation, which in turn impacts the CNS (27). Secondly, poor nutrition is associated with cell and tissue damage, which triggers Toll-like receptors activation and central immune signaling events (28). Thirdly, poor nutrition can change gut-microbiota composition that results in systemic inflammation (29). Additionally, the high levels of anxiety, depression and chronic pain in malnourished individuals, which are all risk factors for poor postoperative pain management, might contribute to postoperative inadequate analgesia as well (30–32). In addition, female gender was also identified as a predictor of postoperative inadequate analgesia and women experienced worse postoperative pain relief than men. These results agree with our previous observation in orthopedic patients, which shows that female patients represented severer postoperative pain than male patients (20). Furthermore, high nutritional risk as a predictor for postoperative inadequate analgesia was more prominent in female patients and early elderly patients. Collectively, based on the current study, a better analgesic should be considered for postoperative pain management in elderly patients with high nutritional risk, especially in female patients and early elderly patients.

Next, we determined the optimal value of GNRI using ROC curve analysis. GNRI of 88 was identified as an optimal cut-off value for postoperative inadequate analgesia, which was lower than the original cut-off value of GNRI. The original cut-off value was calculated by using the cut-off values for weight loss and albumin in the elderly (weight/WLo = 0.95 and albumin = 35 g/L) (9). The results in the current study indicate that the optimal cut-off value of GNRI might be different in different conditions. However, the trend is the same that the value of GNRI is lower, the nutrition-related risk is higher. Furthermore, the new cut-off value was more specific but less sensitive than the original cut-off value. Given that inaccurate diagnosis of malnutrition will cause unnecessary treatment and increase the cost of hospitalization, the higher specificity of the new cut-off value is extremely important in nutritional assessment (33). Collectively, GNRI < 88 could be used as a criterion to screen patients' nutrition-related risk of postoperative inadequate analgesia in clinical practice. Early detection of nutrition-related risk before surgeries might contribute to timely nutritional care and the consequent improved postoperative pain outcomes.

Some risk factors for poor postoperative acute pain outcome were identified in previous studies. A large prospective international multicenter database analysis determined 8 risk factors for severe postoperative pain (numeric rating scale ≥ 7 points) (31). Another meta-analysis of 33 articles identified 9 predictors of poor postoperative pain management (32). However, both studies included more than 50,000 patients with appreciable heterogeneity. Thus, a particular predictor identified might not fit for certain specialties like elderly patients. Through the present analysis focusing on elderly patients, some predictors (e.g., female gender) but not others (such as younger age and higher body mass index) were confirmed. Furthermore, GNRI was added as a novel predictor. These predictors might be useful to stratify inadequate analgesia risk, develop population-specific clinical care pathways and improve pain outcomes in elderly patients. In elderly patients with high nutritional risk, standardized assessment of nutritional status, adequate implementation of nutritional support and aggressive treatment of postoperative pain should be considered.

The current study has several limitations. Firstly, the results of the current study are based on postoperative data of one single university hospital. However, the single center data may have the strength because of standard treatment, such as similar anesthesia and postoperative analgesic management. Secondly, this is a retrospective cohort study, which has relatively poor control over the exposure factor, covariates, and potential confounders. Therefore, the data obtained in the current study should be cautiously interpreted. Further prospective randomized trials to verify these results are warranted in the future. Thirdly, the new suggested cut-off value of GNRI had relative low sensitivity and specificity. Further studies are needed to evaluate its validity in larger populations. Finally, the generalizability of this study is limited to elderly patients with gastrointestinal surgery. Whether this conclusion is appropriate for patients undergoing other surgeries needs further analysis and studies in the future.

In conclusion, this retrospective cohort study demonstrated that the majority of the hospitalized elderly patients undergoing gastrointestinal surgeries had high nutrition-related risk using GNRI. In addition, lower GNRI was association with poor postoperative pain outcomes, which indicated the need for early nutritional evaluation and supplementation in elderly patients undergoing gastrointestinal surgeries.

## Data Availability Statement

The datasets generated for this study are available on request to the corresponding author.

## Ethics Statement

The studies involving human participants were reviewed and approved by the Ethic Committee of Tongji Hospital, Tongji Medical College, Huazhong University of Science and Technology (TJ-IRB20190403). The ethics committee waived the requirement of written informed consent for participation.

## Author Contributions

HZ and XZ contributed to the conception of the idea and the study design. HZ prepared the data set, performed the analysis, and wrote the manuscript. GD contributed to analysis and interpretation of data. SS and XZ provided intellectual inputs for the project and critical comments on the manuscript. All authors discussed the results and commented on the manuscript.

## Conflict of Interest

The authors declare that the research was conducted in the absence of any commercial or financial relationships that could be construed as a potential conflict of interest.

## Publisher's Note

All claims expressed in this article are solely those of the authors and do not necessarily represent those of their affiliated organizations, or those of the publisher, the editors and the reviewers. Any product that may be evaluated in this article, or claim that may be made by its manufacturer, is not guaranteed or endorsed by the publisher.
